# The Hospital. Nursing Section

**Published:** 1903-06-20

**Authors:** 


### The Hospital.

### Nursing Section.

Contributions for this Section of "THE HOSPITAL" should be addressed to the EDITOR, "THE HOSPITAL" NURSING SECTION, 28 & 29 Southampton Street, Strand, London, W.C.

No. 873.—VOL. XXXIV. *SATURDAY, JUNE* 20, 1903.

### Notes on News from the Nursing World.

### ROYAL TRIBUTES TO HOSPITAL NURSES.

ON the occasion of the visit of the King and Queen to the London Hospital last week, his Majesty, in declaring the new buildings open, expressed his deep feeling of gratitude to the hospital, which at the time of his severe illness provided him with such a distinguished surgeon as Sir Frederick Treves, with such an anæsthetist as Dr. Hewitt, and with two such nurses as Nurses Haines and Tarr, "whose unceasing attention," he added, "I cannot too highly praise." These two nurses had also the honour of accompanying their Majesties on the tour of inspection. In the section devoted to the Roentgen-rays treatment, the Queen presented badges to twelve nurses who had served in the South African war. The Hon. Sydney Holland presented the nurses to her Majesty, who said, "I am glad to see my nurses back. I was confident when I sent them out that they would do honour to the training they have received from Miss Lückes at this hospital, and I now wish to give each of them a little memento of their service abroad for me." Her Majesty then pinned upon the breast of each nurse a white enamel cross with a crown and the cypher "A"—a badge similar to that given to the nurses in the hospital ship. The recipients of this distinction were Miss Becker, R.R.C., now principal matron of Queen Alexandra's Imperial Military Nursing Service; Miss McCarthy, R.R.C., matron at the Woolwich Hospital; and Misses Greenham, R.R.C., A. Thomas, V. P. Squires, E. Fry, E. Baldrey, L. Humphreys, G. A. Thorpe, N. E. Tate, H. Parminter, and N. R. Holloway.

### QUEEN'S NURSES FOR CONNEMARA.

As a result of the appeal of Lady Dudley for funds to enable her to establish and maintain Jubilee nurses in some of the poorest and most congested districts in Ireland, arrangements have been made for the establishment of a nursing station in Connemara. The dispensary residence at Benlodangar has been vacated in order to provide accommodation for the nursing staff whom Lady Dudley is prepared to supply; and, as showing the local feeling towards the movement, it may be added that the Oughterard Board of Guardians have approved the proposed arrangement.

### THE WAR OFFICE AND NURSES.

THE answer given in the House of Commons last week by the Secretary of State for War to the question of Sir John Leng respecting the withdrawal from nurses of the privilege of wearing clasps with their South African medals is unsatisfactory. Mr. Brodrick's reply was: "The grant of clasps was confined to those persons who were either combatants or who intended to go under fire. The privilege cannot, therefore, be extended to the nurses." In point of fact, Mr. Brodrick merely evaded the question. There would have been no grievance if the nurses had not in the first instance been permitted to wear clasps with their medals. The essence of the complaint is that the privilege having been granted it has been taken away. We can only conclude that it was conceded by mistake, and mistakes of that kind are not creditable to the Department for which Mr. Brodrick is responsible.

### BEDFORD COLLEGE AND HOSPITAL ECONOMICS.

IN the early part of this year we advocated the formation of a course in Hospital Economics at Bedford College. Our proposal was that that course should be arranged by the college authorities in consultation with the leaders of nursing in England. Otherwise the course must fail to do the work required. We are informed that the college authorities have not appreciated this point, owing to their lack of knowledge of nursing politics, and the college is at present being wooed by the Matrons' Council. We hope that the Bedford College authorities will avoid the rock on to which they are drifting. Should an attempt be made to approach the hospital matrons of London through the Matrons' Council, such a step must infallibly mean the non-co-operation of the training schools as a body, and the failure of the Bedford College scheme. If the Council of Bedford College will glance at the composition of the Matrons' Council they will notice the absence of the names of the matrons of the London Hospital, King's College Hospital, St. Thomas's and Westminster Hospitals, and indeed of the real leaders and controllers of nursing education in England to-day. It is essential to success that Bedford College should avoid falling into the hands of a clique, and we are confident that its council will be prudent enough to approach the matrons of the great London hospitals direct as the plan best calculated to ensure the success of their course of lectures on Hospital Economics.

### THE SALARY QUESTION IN THE CROWN COLONIES.

A FEW months ago one of our correspondents in the Far East called attention to the new scheme of the Government for paying Colonial servants in sterling, by which arrangement she contended that nurses who had been working in Government Colonial hospitals for several years would find their salaries reduced by several pounds per annum. By the last mail the same correspondent sends a letter, which we publish to-day, announcing the decision of the authorities. This decision has reference only to nursing sisters who have been employed before 1901, and does not in any way affect sisters who have joined recently.

### CONFERENCE ON WORKHOUSE NURSING.

THE decision of the President of the Local Government Board with regard to the report of the Departmental Committee on Nursing, which was appointed at his instance, will perhaps be quickened by the proceedings at the inspectors' conference which is to be held under the auspices of the Workhouse Nursing Association at 10 Great George Street, Westminster, on Monday, June 29, at 3 o'clock P.M. The chair will be taken by Mr. J. G. Talbot, M.P. The discussion, after a brief introductory statement by Miss Wilson, will be opened by Miss Amy Hughes, and speeches will be delivered by Miss Louisa Twining, Miss Gibson, Miss Brodie-Hall, Miss C. J. Wood, Mr. H. Bonham-Carter, and Mr. J. Heywood Johnstone, M.P. If Mr. Long can spare an hour he might spend it very profitably in listening to the criticisms of experts on the departmental report.

### A HUNDRED APPLICATIONS FOR SIX VACANCIES.

FOR six vacancies at King's College Hospital this month there were 100 applications from would-be probationers. Of course, the reputation of King's justly stands very high in the nursing world, but it would be a mistake to attribute to that fact only the number of candidates eager to obtain admission to the training school. It indicates that the public are much better informed than they used to be, and that they have learnt not to treat seriously the vague statements which are often made about hospital nurses being overworked and underfed.

### A DRAWING-ROOM MEETING AT SURREY HOUSE.

BY the invitation of Lady Battersea, a drawing-room meeting for members of the nursing profession will be held at Surrey House, Marble Arch, on Thursday, July 2nd. Sir Thomas Barlow and others have promised to speak on the occasion, and music and refreshments will complete the programme of the entertainment, which will last from 3.30 to 6.30. Although, of course, no one will be admitted without an invitation, any nurse who would like to be present and writes for a card to the Hon. Secretary, Miss Hilda Dillon, 47 Oakley Street, Chelsea, S.W., will receive one. Lady Battersea is anxious that as many nurses as possible should avail themselves of the opportunity of spending an afternoon in her beautiful home.

### A USEFUL MOVEMENT.

THE objects of the Rural Midwives' Association, whose meeting on Monday is reported in another column, merit the warmest commendation. They are to train and supply midwives for rural and provincial districts where required, as well as for associations wishing to add them to their staff, and likewise to give assistance in training in special cases. The association has a strong executive committee, with Mrs. Heywood Johnstone as chairman, and the medical honorary members include several eminent physicians.

### AN EXPERIMENT AT PRETORIA.

IN contrast to the success of the Johannesburg Nurses' Co-operation, we hear that the attempt of a few enterprising sisters to establish a nursing home at Pretoria has so far been a failure. At the outset they were handicapped by having to pay the enormous rent of £900 a year, and prices are still so high, eggs for example being 1s. each, that they cannot make two ends meet. Yet there is an acknowledged want of trained nurses in the town, and the conditions seemed favourable when the sisters decided to risk their money in the undertaking. Their disappointment is another warning to nurses who are in appointments at home not to think of rushing off to South Africa unless work is assured them.

### IS CROQUET NECESSARY FOR THE WELL-BEING OF NURSES?

THE Southampton Guardians have decided to purchase a croquet set for the use of the nurses at the infirmary. They had previously consulted the Local Government Board as to the legality of the expenditure, and received in reply an intimation that the question turned upon whether or no the articles could be regarded "as necessary for the well-being of the nurses," and were such as they could not be expected to provide for themselves. It was added that if the Guardians determined to provide the croquet set, it would rest with the district auditor in the first instance to deal with any question which might arise as to the reasonableness of the expenditure incurred. As to the point whether croquet is necessary for the well-being of nurses, we cannot pronounce absolutely in the affirmative. But all recreation that tempts nurses to spend their off-duty time out of doors is certainly desirable; and as croquet is a game which can be played without weariness, it is especially suitable for them. It seems to us, however, that there is a better way open to the Southampton Guardians than running the risk of being surcharged by the district auditor. An adequate set of croquet may be purchased for £2 or £3, and as we know that there are several men of substance on the Southampton Board, we suggest that they could not show their appreciation of the work of their nurses more gracefully than by subscribing for it among themselves.

### THE HOLIDAY QUESTION AT BRISTOL.

THE Bristol Board of Guardians are to be congratulated upon the fact that they have agreed, by a substantial majority, to extend the annual holiday of the nurses in their employment to three weeks. Hitherto the nurses have only had a fortnight, which, as some of the guardians pointed out, is less time than that allowed by the principal boards in the country. There has been considerable difficulty in obtaining nurses at Bristol, owing, as it was contended on one side, to the long hours and short holiday. On the other hand, it was urged that the reason for the difficulty was that nurses were "pampered so much,"—we conclude, elsewhere. If that be the case, it may be worth the while of the Bristol Board to do a little "pampering" on their own account in order to secure a full staff; and the concession of three weeks' holiday may have the desired effect of increasing the number of candidates for vacant appointments.

### GRATITUDE IN DISTRESS.

A few days ago an accident case was admitted into the Norwood Cottage Hospital. The man subsequently died and an inquest was held. The widow gave evidence, and at the close of the inquiry her aged father said he wished to publicly express his gratitude and appreciation of the kindness of the matron and nursing staff. His daughter suffered badly from heart disease, and but for the sympathy and consolation of the matron he was sure that the shock to her on reaching the hospital would have proved fatal. Such sympathy he had never experienced before and should never forget. When the jury had left, the matron, Miss M. K. Clark, and a nurse entered the room, and taking charge of the distressed widow led her at her own strongly expressed wish to the mortuary to see the body.

### DISTRICT NURSING AT MATLOCK.

At the annual meeting of the Matlock District Nursing Association several speakers urged that the appointment of a second nurse was necessary, and as the single nurse employed by the organisation paid nearly 3,000 visits last year, it is obvious that she has more than enough to do. But the objection was urged that funds would not justify the association in providing her with a colleague. We notice, however, on referring to the balance-sheet, that the total receipts were £218 14s., and although £87 19s. 11d. of this consisted of an amount brought forward, there should scarcely in the circumstances be any difficulty about finding the salary for a second nurse, if she is, as it appears to be the case, badly needed. The expenses include "housekeeper's salary," but the amount is not mentioned. District nurses should be adequately paid and comfortably provided for. At the same time it is possible to err on the side of extravagance, and we think that with proper economy in the management and a little energy in collecting subscriptions, the Matlock Association would be justified in making an addition to the staff.

### THE APPEAL OF A WELSH TRAINING SCHOOL.

The medical officer of the Merthyr Tydvil Workhouse has addressed a letter to the secretary of the Local Government Board urging the claims of the Union Infirmary to be recognised as a major training-school. He avers that with the single exception that there is no resident medical officer, the Merthyr Infirmary contains all the essentials of a training school as set out in the report of the departmental committee, and he contends that this is to a great extent met by the fact that he and his assistant at his private house and surgery are in direct telephonic communication with every block and charge nurse in the infirmary, and are only three minutes' walk from the workhouse. Dr. Ward goes on to cite certain facts in support of his plea, which are worthy of consideration. The Merthyr Union Infirmary was one of the first in the country to start training probationers as workhouse nurses, and has trained 25 probationers who have obtained their certificates at the end of their three years' training, after being examined by three medical men and the superintendent nurse in medical and surgical nursing. The nurses, Dr. Ward says, are now much sought after by nursing institutes and workhouses in Wales ; and the infirmary, he points out, contains 129 beds, as well as 66 beds in the hospital at the house for overflow cases, where there are night and day nurses on constant duty. He adds that the infirmary is well equipped as a modern hospital, has an up-to-date operating-theatre, a marquee for the outdoor treatment of pulmonary tuberculosis from May to the end of October, *x*-ray apparatus for diagnostic purposes, as well as *x*-ray light treatment of lupus, and a maternity department. In these circumstances we can well understand that both the medical officer and the Guardians are much disturbed lest the infirmary should not be recognised as a major training-school. But the Local Government Board must, of course, adhere to regulations which are founded upon well-defined principles, even though, as in this instance, there may be reasons for making an exception.

### UNFOUNDED CHARGE AGAINST NURSES.

The Ross Board of Guardians have been engaged in an inquiry into the allegations of a girl as to her treatment by the nurses and doctor when she was in the Ross Workhouse Infirmary. A few hours before her death the girl told her mother that she had been given new bed-ticking to sew, and that stale meat and margarine were provided for her to eat. Facts, however, showed that there had been no new ticking in the workhouse for more than a year, and the house surgeon was able to testify that the only sewing she was given consisted of some light articles which the patient asked for to keep her from "moping." As to her food, he stated that she had whatever she liked to eat, and that her diet consisted principally of eggs, milk, and port wine. She had told the house surgeon that she was perfectly satisfied with the treatment which she had received. The Board considered that there was no cause for complaint either against the doctor or the nurses.

### WARWICK NURSING ASSOCIATION

At the annual meeting of the Warwick Nursing Association, the Countess of Warwick in the chair, a very satisfactory report was read and adopted. The number of cases nursed was 216 and of visits paid 6,914 ; while the balance in hand at the end of the year was £24. The happy financial result was largely due to the ball which yielded £66, and a donation from the Warwickshire Hunt Club of £33. The friendly societies likewise contributed £7. Lady Warwick, however, urged upon the subscribers the necessity of not relaxing their efforts, and dwelt upon the importance of maintaining a large number of small subscriptions.

### THE SCHOOL OF DISTRICT MIDWIVES.

Miss Gregory's meeting in support of this proposed school is being held at 3 Grosvenor Place, S.W., this (Thursday) afternoon, at three, as we go to press. The Bishop of Stepney, Mr. Asquith, K.C., M.P., and Mrs. Scharlieb are announced as the principal speakers, and much interest is being displayed as to the line they are likely to take in support of this movement.

### SHORT ITEMS.

The s.s. *Sicilia*, which arrived from South Africa at Southampton on Monday, had on board Nursing Sisters J. F. Farrer and L. M. Monk of the Army Nursing Reserve Service, who return in consequence of the reduction of establishments.—An "At Home and Garden Party" are being held at the West Ham and East London Hospital, Stratford, on Saturday, the 27th inst., from 3 to 6 p.m. Invitations can be obtained from the Matron.

### The Nursing Outlook.

"From magnanimity, all fear above;
From nobler recompense, above applause,
Which owes to man's short outlook all its charm."

### THE NURSE'S DINNER TABLE.

Every matron and superintendent is bothered at intervals with complaints about the food provided for the nurses and staff; sometimes these complaints are well grounded, but generally they arise from the folly of a monotonous diet. In the stir and hurry of hospital work it is only too natural for the matron who has fixed up a lengthy weekly dinner bill to let it alone from month's end to month's end, and to feel aggrieved if her judgment is questioned. But a healthy diet must be a varied diet. At the meeting of the Sanitary Institute at Manchester last year a dear, prim old lady got up and lengthily explained the system of feeding the children at a certain school, and how carefully the food was weighed and adjusted to give the right proportion of albuminates and carbo-hydrates. She mentioned incidentally that the children had porridge for breakfast all the year round. Then Dr. Newsholme, of Brighton, arose and said, in effect, that he expected such children would be mentally stodgy, for you could not get varied work and varied thought from children fed always on a monotonous diet.

So we would put in a plea with matrons to provide not only a plentiful supply of nutritious food for their nurses, but also a pleasant amount of variety and of seasonable dishes. As a rule the money allowance is sufficient, it is only a little more time and thought that are needed. There is a good old housekeeping rule that gives 10s. a head a week for food. The guardians of most infirmaries allow this for the staff and nurses, and the London School Board allows it for the officers in its residential schools. It is a very ample allowance indeed for big institutions, especially as most of the food is bought at contract prices. But still at one infirmary we know where this allowance is granted, the nurses only have pudding once a week, and the eggs supplied for breakfast are always sent up boiled. Now there are at least a dozen simple puddings that can be cooked and supplied in quantities; and there are at least half a dozen simple ways of cooking eggs. Then plain cakes are quite as cheap as bread and butter, and jam is cheaper than butter, and yet we have heard of a big hospital which gives its nurses bread and butter for tea from one year's end to another. Again fish is cheaper than meat, and twice a week at least should make its appearance at the nurses' table. Vegetables are often stinted, and fruit is never supplied at all. The whole world is our fruit-market now, and there is no difficulty in arranging for the supply of oranges, apples, bananas and figs for the whole year round. It is not only that constant change of food is healthy, but it takes away from the dreary institution taint that is too apt to fall on a big hospital or infirmary. And if the varied food has for sauce varied conversation—if the matron dines with her nurses and makes talking and entertaining an art, then still more value is derived from the meal. In a great institution the matron cannot always do this, but she can do it sufficiently often to set an example, and in her absence a sister can take the head of the table and act as hostess and try and keep things bright.

Poor "matron"! what a lot we demand and expect of her! For on the other side she has her committee to consider. Quite lately a Board of Guardians in the North of England heard of a matron who managed her allowance so economically that she was able to let the nurses always have fresh butter, and occasionally poultry. Instead of praising her, they censured her, and said best Dorset and boiled beef were proper food for nurses, and they did not want the ratepayers' money spent on "dainties." That we believe is a unique case and will not, we hope, dissuade other matrons from seeking to alleviate the stolidity and sameness of institution meals. So long as the matron keeps within the allowance she ought to be encouraged towards individuality. The more solid portions of the diet—the meat, the bread, and so on—must of course come from the contractors in measured portions, but there ought always to be a margin for things which are cheap and seasonable. There are times when the market is flooded with tomatoes or celery, and yet these wholesome vegetables seldom appear on the nurses' table. There are hot days in summer when cold meat and salad is the only appetising food; and yet the hot joint and boiled potatoes are served with monotonous thoughtlessness. With some superintendents this is all part of the "training." They hold the old theory we were taught in the nursery that it is vulgar to consider food, and that nurses ought to take what they are given and be thankful. They remind us that in Plato's "Banquet" the food is never once mentioned, and that we ought "to eat to live, not live to eat." All of which is true, and we heartily agree that there is no room for the chronic grumbler in the nursing world and that the sooner she is dismissed the better. Yet prevention is better than cure, and the occasional grumble might often be prevented if matrons would recognise more widely that a suitable diet is a seasonable diet, and that monotony makes even good meals unpalatable.

### Lectures on Medical and Surgical Nursing.

By John Hopkins, F.R.C.S., Medical Superintendent of the Central London Sick Asylum District Asylums, Cleveland Street, W., and Hendon, N.W.

### LECTURE X.—DISEASES OF THE RESPIRATORY ORGANS.

When inflammation attacks the air passages it does not, as a rule, affect them throughout their length. Some one part is mainly or exclusively the seat of the disease. Thus we have laryngitis, tracheitis, bronchitis, capillary bronchitis (in which the smallest bronchi are inflamed), and pneumonia, which mainly affects the alveoli. When the connective tissues of the lungs are diseased the condition is called phthisis. The deposition of tubercle you are already acquainted with. There may be a deposition of dust from stone, iron, etc., which, drawn in with the breath, is taken up by the living cells and conveyed from the alveoli and bronchi into the connective tissues surrounding them. This dust may be accumulated in large quantity and give rise to the formation of fibrous tissue in abundance which is organised from the inflammatory effusion that surrounds the foreign particles, and which shrinks as the fibrous tissue of scars from burns shrinks, and limits the expansile power of the lungs at the same time dilating the bronchi. This is known as fibroid phthisis.

In *pleurisy* there may be a scanty secretion of inflammatory products into the pleural cavity from which a coagulum of lymph is deposited, and, becoming organised, binds the lung to the chest wall. This is called *dry pleurisy*. In other cases there is a very abundant secretion of this serum into the pleural cavity, which compresses the lung and needs removal by the aspirator.

In empyema there is pus in the pleural cavity, which will make its way out through a bronchus by a process of ulceration, or through the chest wall, if not let out by operation. When it escapes through a bronchus, air takes its place and there is a condition of *pyopneumo-thorax*. Accompanying inflammation or independent of it, there may be muscular spasm. When there is laryngeal spasm there is danger of sudden death from want of air. When there is bronchial spasm the inspiratory dyspnœa may be extreme and alarming. This latter condition is known as *asthma*.

*Dyspnœa* is a common feature of disease of the respiratory tract. Any obstruction limiting the entry of air causes the patient to breathe with increased exertion. The obstruction may be in the nares, pharynx, fauces, or any part of the air tubes. In children this causes the chest walls to fall in and there may be a resulting deformity. A common cause of this is obstruction by adenoids and enlarged tonsils. If the alveoli be filled with the inflammatory products of pneumonia, which often affects as much as the half of a lung, or, if the lung be compressed, there is a corresponding dyspnœa. Breathlessness is not necessarily due to disease of the lungs or respiratory tract. Failure of the heart to pump the blood through the lungs, or failure of the blood to undergo proper aeration will also cause it. Anything limiting the supply of air to the lungs, such as obstruction of air-passages or limited lung capacity, calls for abundant pure air. If this be supplied quite fresh, breathed neither by the patient lying in too close a steam tent, nor by others in an ill-ventilated apartment, the first step has been taken to relieve the patient. When the difficulty of breathing prevents the patient lying down and sleep is interfered with, there is an additional danger to the health.

*Cough* is a provision of nature for the purpose of expelling irritant mucus, pus and blood from the lungs. As long as there is anything to be expectorated the cough is an excellent thing. Inflammation in an early stage will excite a dry cough, and the mere act of coughing may irritate the larynx and thus supply a cause for its continuance when there is no more mucus to be expelled. Under such circumstances a sedative is useful, but care must be taken not to check a cough that is doing good.

*Expectoration.*—The products of inflammation may be clear mucus, or opaque and yellow from the presence of abundance of cells or fluid pus. From a gangrenous lung, a pyopneumo thorax, or a dilated bronchus, there may come a fœtid expectoration. When a person coughs the air is sprayed with fine particles of saliva and mucus, which in the case of consumption carry with them the bacilli of tubercle. This spray is carried, according to the size of the particle, for varying distances: the smallest may float some yards before settling down. Hence walls and furniture become covered, and should be regularly cleaned, the making of dust being avoided in the process. The expectoration should be disinfected as well as the utensils used to hold the sputum.

*Hæmoptysis.*—The spitting of blood in any quantity becomes alarming to the patient and to those standing by. Sudden death may be the result. When the blood is in such quantity as to cause alarm, the patient attempts to suppress the cough, hoping to check the hæmorrhage. In this he must be discouraged, and assured that the best way to stop the bleeding is to breathe and cough as nature prompts him, for by that means the blood in the tubes will be the more quickly removed, and thus the breathing will become easier. Any straining effort will only tend to increase the bleeding. The care of pneumonia needs special reference. In this disease the beginning of convalescence is by *crisis*. The temperature suddenly falls, generally somewhere between the fifth and eighth days, and the patient then rapidly improves. Any delay in the appearance of this crisis causes fear that the case may go wrong, and if it does not occur before 14 days have elapsed there is something more the matter with the patient. The severity of the case and the prospects of recovery can be in part estimated by the expectoration and the frequency of the pulse and respiration, so the sputa which are rusty generally should be kept for inspection, and the pulse and respiration recorded on a chart every four hours.

Sometimes pneumonia is accompanied by delirium or sleeplessness, and to procure sleep drugs are administered that need to be repeated at intervals, sometimes frequently, and their effect has to be carefully watched. A nurse that can watch a patient's pulse and intelligently interpret the changes in it would be able to render great service. To become efficient in this constant practice is necessary. I would recommend you always to feel the pulse of those you are nursing in order to acquire the requisite skill. Digitalis is much used in cases of delirium and in pneumonia, and requires pushing often in large doses, but this can only be done when the patient is kept under close observation, for it alters the pulse, causing fuller beats and at the same time lowering the rate. Sleep can only be obtained when this effect is produced, and the object in watching the pulse is to know when the dose shall be repeated or when the drug shall be stopped. It might be a fatal mistake to continue the administration of a dose of digitalis longer than was absolutely necessary.

### Colonial Nursing Association.

"NURSES MORE IMPORTANT IN TROPICAL COUNTRIES THAN DOCTORS!"

By the kind invitation of Field-Marshal Sir Henry and Lady Norman, the annual meeting of the Colonial Nursing Association was held at the Royal Hospital, Chelsea, on Thursday last week. Princess Henry of Battenberg (Patroness of the Association) was present, and in spite of the wet afternoon there was a good attendance, including Mrs. Chamberlain, Lady Balfour of Burleigh, Mrs. Francis Piggott, Lady Ommanney, Lady Mitchell, and Mrs. Ernest Debenham.

The chair was taken by the Earl of Westmeath in the absence through illness of Earl Grey, the President of the Association. In his opening remarks Lord Westmeath said that two names stood forth in the history of this Association, those of Mrs. Piggott, who after having had much experience of the want of trained nurses in distant parts, threw herself into the work of supplying that want, and Mrs. Chamberlain, who had also taken up the work with a zeal and energy of which it was impossible to speak too highly. It was due very largely to Mrs. Chamberlain's endeavours that they were now in their present fairly satisfactory financial position. The report showed that the year's work was distinctly good and progressive. The number of nurses sent out was 35 as against 40 in 1902, but this apparent falling-off was explained by the fact that the nurses were engaged under agreements of two or five years, and several agreements terminated in 1902. The total number of nurses at work was 97, as against 88 in 1902, of whom 73 were employed by Government and 24 privately.

The Earl of Selborne moved the adoption of the report and observed that regarding the matter simply from a humanitarian point of view, it must make an enormous difference to the men who went out as pioneers to the ends of the earth, to think that they had a chance of being nursed by a woman if sickness overtook them. These men carried with them the whole honour and credit of England. Therefore, in the work of Empire-building, what was wanted was not that they should send second-rate men, much less wastrels, but their very best. That was the class of men they were sending out now, but in order to ensure the continuance of the supply of this class they must do all that they could to lessen the risks to health by disease. The parents of the young fellows they wanted would naturally shrink from allowing their sons to face such risks. If they could lessen the chances of disease, and the fatal endings of sickness, they would also lessen the opposition to the best young men being sent out to tropical climates. This nursing movement was therefore an Empire-building movement, and one most calculated to improve the conditions of life under which these young men lived.

Sir M. Mitchell-Thomson, representing the Scottish branch, seconded the adoption of the report.

Sir H. Johnston, speaking in support of the motion, said that after many years' residence in Central Africa he had arrived at the conclusion that nurses were more important in tropical countries than doctors, and that there should be five nurses for every doctor. The doctor could only diagnose the disease and prescribe remedies, but often the only chance of restoration to health lay in careful nursing. Since this Association had begun its work residence in tropical countries had been shorn of half its terrors and the health of Government officials had improved wonderfully. He confessed that he had formerly feared lest women should prove encumbrances in Africa, but he had learnt that they were the direct opposite. Women in tropical countries enjoyed better health than men, and they rendered immense services to the white men by enabling them to enjoy the benefits of civilised society. Life in distant lands improved when women took their due share in the development of the country. It had been stated that the one unlooked for result of the scheme was that the nurses not infrequently married, but that, in his opinion, was a consummation devoutly to be wished for. Where could the white men find wives better trained than the nurses sent out by this Association?

Sir Henry Burdett said that it gave him great pleasure to hear from Sir H. Johnston that the influence of the English women they sent out into British colonies was wholly good, and that they not only had a civilising effect on the native population, but had also brought with them hope to the white men of surviving the maladies of the country. It was only fair to remember that they owed the Colonial Nursing Association to a woman, who had given up her time and energy to the work; he was alluding, of course, to Mrs. Piggott. They were also most fortunate in having the support of Mrs. Chamberlain and her husband, who had done so much to help forward Empire building. The Association had now existed eight years, and during those years it had made steady and continuous progress, but there was much yet to be done. The report showed that the nurses had gone into nearly twenty colonies, but there were many parts where nurses were sadly wanted, but where, for lack of resources to pay the nurses, they could not be sent. He drew attention to the fact that although they now had £5,000 invested, which was sufficient for their present work, yet it was insufficient for the work that lay before them. Of the nurses employed by the Association two-thirds were in Government employ and only one-third were employed privately, but he would prefer to see the figures reversed.

Sir Henry then suggested that, following out the idea of the Queen's nurses, they might get together a small committee of men who had done their work as Empire-builders and had come home to rest, but who would be glad to identify themselves with their old colonies and might help, both by getting money together to defray the initial cost of sending the nurses, and also by furnishing the Association with information as to the needs of their Colonies. With this information it might be possible to obtain a certain amount of assistance from the local Governments or from the Colonial Office. In doing this work they would only be following the excellent precedent and splendid example set them by Mr. Chamberlain, of whom history would record that he was so much a man that though burdened with the heaviest responsibility a Colonial Secretary had ever had to bear, he still had heart and energy to recognise that the health of the white man who represented the Government was essential to the success of the Empire. They should always feel grateful to Mr. Chamberlain for having taken up the two questions of tropical medicine and colonial nursing. Every part of the British Dominions, no matter how far distant, nor how small, ought to be provided with a nurse, through the Association, and then whatever the cost, he affirmed it to be his deep conviction that the money would be forthcoming.

Sir Ralph Moor, who proposed the election of officers, offered the suggestion that the remuneration of the nurses should be increased, and that they should not be required to put in the whole of their time for pension—a period of 18 years—in the same colony.

COLONIAL NURSING ASSOCIATION—*Continued.*

Major Ronald Ross supported the motion, and said he was highly satisfied with the interest the nurses sent to the Tropical School of Medicine took in their work, and it had now been decided that they should have the full course given to fully qualified men. He said he would like to see many more of them. This Association was to his knowledge not only desirable but absolutely necessary, if the country was to do its duty to those sent out to tropical countries. He, too, had a suggestion to make, which was that the Association should not content itself with sending out nurses to hospitals, but should go further and send ladies to assist in the work of sanitation.

Lord Westmeath proposed a vote of thanks to Princess Henry of Battenberg for her presence, and said that the Association owed much of its success to the unfailing interest her Royal Highness had always taken in the work.

Votes of thanks were also passed to Sir Henry and Lady Norman and to Lord Westmeath.

### Rural Midwives' Association.

A drawing-room meeting in the interest of the Rural Midwives' Association was held at 3 Grosvenor Place, by permission of Lady Esther Smith, on Monday last. It was clear that the promoters of this Association had succeeded in arousing the sympathetic interest of a large circle of friends, for there was a crowded meeting in spite of the incessant rain. Among the company present were the Duchess of Montrose, the Countess of Verulam, Lady Ulrica Thynne, Lady Elizabeth Byng, Lady Edith Fox Pitt, Lady Camoys, Lady Fremantle, Lady Bigge, Lady Grantham, Lady Hannen, Lady Muntz, and Mrs. Pitt Rivers.

The chair was taken by the Countess of Ancaster. Mr. Heywood Johnstone in a short introductory speech said that this Association was the outcome of a very satisfactory meeting held on March 3rd, and they were now gathered together to relate what had since been done, what had yet to be done, what the need was, and how the Association proposed to meet it.

Dr. Percy Boulton then pointed out that, by the enactments of the Midwives Bill passed in 1902, uncertificated midwives would be prohibited in a few years' time from pursuing their profession. It was his opinion that the supply of trained midwives for towns would be ample, but this would not be the case in scattered rural districts, where it would be most difficult to get the right class of women sufficiently educated to discharge their duties adequately, yet humble enough to take their place in the cottages and act the part of friendly neighbour. It was to supply these women that this Association had been formed for the training of midwives.

Sir Michael Foster, M.P., said that in the great majority of cases of childbirth medical aid was not necessary; all that was needed was ordinary care on the part of the midwife and a cleanliness that must be beyond reproach. But it must not be overlooked that in certain cases of childbirth a patient might at any moment be in imminent peril of life and a trained midwife must be able to grasp the imminence of the danger and be prepared to call in a doctor. A certain amount of training was therefore necessary, but too high training was not advisable, as a midwife might then fancy herself able to accomplish what ought to be left to a doctor. Another drawback likely to result from too much training was its rendering a midwife above her place and preventing her from making herself at home in a poor cottage and doing the work of the head of the house.

Mrs. Heywood Johnstone observed that this organisation was prepared to undertake the training of young women at existing institutions who agreed to serve under the Association as cottage midwives, in most cases for a period of three years. Several institutions had very kindly offered to reduce their fees, one even offering a free training. For the benefit of the poorer country districts which were unable to pay for the training of a midwife, they were anxious to establish a system of grants whereby a considerable portion of the expense might be met. They had already started the training of several midwives, and from Hull and Winchester they received excellent reports of their progress. Thirty probationers were anxious to be trained, and they had established six centres. What was needed was funds, both for training and for the supply of grants.

Mrs. Hobhouse stated that the Association had two objects in view, firstly, the establishment of new branches to provide that in those places where there was no nurse there should be a nurse, and that nurse should be a midwife, and secondly, that in those places where they already had nurses, they should have a midwife added to their staff. The training for a midwife would cost £15 15s. to non-subscribers; subscribers of £5 might nominate two pupils a year at a reduced fee of £12 12s. No midwives were to be permitted to give their services free. The fees of a medical man, if his services were required, were to be guaranteed by the Association and not by the patient.

A vote of thanks to Lady Ancaster and to Lady Esther Smith were subsequently passed, and Lady Ancaster, in responding, expressed her hearty approval of the objects of the Association.

### Everybody's Opinion.

### THE STERLING SALARY SCHEME ULTIMATUM.

"An Occasional Correspondent," writing from the Far East, says: At last the fiat has gone forth and the terms for the future salaries of all colonial servants under agreements have been fixed and approved by the Home Government. In its original standing, the new salary scheme provided for the nurses' maximum to be reached after their sixth year of service by which they would have lost, as compared with their present stipend, an annual sum of £32, plus the cost of fuel and light. Now a concession has been made, by which their maximum salary commences at the end of five years, subjecting them to an annual loss of £12, plus light and fuel. Is this just treatment? Of course there are alternatives, three of which occur to me at the moment. (1) They must accept a reduced fee for services which are assuredly more valuable from the points of experience and acclimatisation; (2) or if in sufficient force they can appeal against this decision; (3) or they can give up their five years' work which would count towards a pension—and go, in which case a free first-class passage to England must be provided for them. Is the Government responsible for this arrangement? Indirectly, yes. Directly, no. The Government or its administrators have to deal with a colossal scheme like this in a wholesale fashion. Employees in their different branches have to be dealt with *en bloc*, and it would naturally be a herculean and impossible task to meet the individual claimant. Still, his case should not be ignored, and the simplest way would be to make the heads of departments responsible for a detailed account of any loss likely to be sustained. The Government is not that close-fisted, hard-hearted vampire it is dubbed, as I can personally testify after some cycles of years spent in its service, but it demands, after a grievance has been stated, an amplified account with well-substantiated facts. Then, if sent through the proper channel, redress may be had.

### THE TRAINED MASSEUSE.

"Elizabeth Manley, a Founder of the Incorporated Society of Trained Masseuses and Chairman of the Board of Examiners," writes: It is suggested in an article in Th

HOSPITAL of May 30th, entitled "The Trained Masseuse," that the Incorporated Society of Trained Masseuses (founded 1894 and incorporated July 1900) may be of use in enlightening the general public on several points relative to the training, status, and qualifications of a masseuse. The founders of the Society gladly take this opportunity of furnishing a few facts which it is hoped may be found useful, and the knowledge of which may help to promote that co-operation and general high standard of proficiency amongst masseuses which is so much to be desired, and which, as your article justly points out, is so greatly wanting in the nursing profession in this country. The article in THE HOSPITAL concludes by saying that "it is for the masseuses themselves to boldly formulate a policy and follow it." May I begin by calling the attention of your readers to the fact that a policy was formulated in 1894, which has been persistently followed ever since? The boldness was shown by the dauntless six who came up for our examination that year; the persistency of the society's policy is evidenced by the fact that the number of candidates at our examination last March was 67. The dates of the examinations in massage held by the Incorporated Society are so arranged as to allow a full term of three months for study and preparation before each examination, irrespective of two months' interval in the summer and an interval of one month in the winter. It is found that the knowledge and efficiency demanded in order to obtain the certificate can be acquired by a student of average intelligence, education, and aptitude in three months, provided that she is able to devote her attention steadily to the subject. A thorough practical knowledge of massage is required and of elementary anatomy and physiology. A knowledge of medical electricity is not exacted, but a large number of candidates attend practical classes for instruction in that subject before or immediately after obtaining their certificate in massage. All teachers of massage who are members of the society, and, we believe, all teachers of good standing who are not members, invariably teach massage on the living subject and on the bare skin, not through clothing, certainly never on "dummies." The pupils of accredited members of the Incorporated Society have the advantage of working under supervision, as soon as proficient, in the wards of a large London infirmary, and in many cases the classes for instruction in massage are attended by out-patients for whom massage has been ordered, and to whom it is a great boon to obtain it gratuitously. All our trainers can participate in this advantage, as the applications from charitable societies and from doctors are numerous and always receive attention. The examinations of the Society are open alike to trained nurses and others, and the doctors who apply to our secretary for a masseuse specify whether or not a trained nurse is required for the particular case. Teachers who "train" pupils in two or three weeks do not send up candidates to our examinations. The Society has not yet found out a method of excluding from the profession all candidates who do not possess the "needful natural gifts" of a masseuse, but teachers are able to do something in this direction by discouraging unsuitable candidates from training. Candidates from any training school are accepted upon due inquiry into references as to character and evidence of training, and we are glad to know that the eminent medical men whom we are proud to claim as our medical patrons are good enough to consider our certificate a satisfactory guarantee of proficiency.

### TRAVEL NOTES AND QUERIES.

PENSIONS IN FLORENCE (Yungfrau).—Here is a list of what you want; but the houses being cheap often do not succeed and are closed, therefore write in good time. Pension Norca, 36 Via Nazionale. 5 francs; Signorina Sartini, 16 Borgo San Jacopo, about the same; also Signora Cicognani, 19 Via de Bardi; Pension Banchi, 54 Viale Principessa Margherita, 6 francs; Pension Bercheilli, 12 Lungarno Acciafoli, 6 francs; Madame Laurent, Via del Presto, don't remember number. I do not think a conducted party would suit you at all, because they race from place to place, only remaining one or two nights in each. The least expensive route is *viâ* Newhaven and Dieppe, Genoa and Pisa. Second class return £8 or thereabouts. So glad the Swiss trip was a success. Many thanks for what you say of articles on Florence. There will be no more as my work is stopped.

### Appointments.

ASTON UNION WORKHOUSE INFIRMARY.—Miss Jessie Elizabeth Price has been appointed charge nurse. She was trained at Salford Union Infirmary.

FEVER HOSPITAL, CAMBRIDGE.—Miss M. Edward has been appointed sister. She was trained at the Royal Infirmary, Sheffield, and for twelve months has been staff nurse at the Borough Fever Hospital, St. Helen's. For the last three months she has been in charge of the small-pox hospital.

FULHAM INFIRMARY.—Miss Gertrude Mary Margaret Evans has been appointed superintendent of night nurses. She was trained at St George's Infirmary, Fulham Road, London, where she afterwards became sister. She has also been assistant nurse at the Gore Farm Fever Hospital.

GRAVESEND HOSPITAL.—Miss L. P. Lessey has been appointed matron. She was trained at the London Hospital, Whitechapel, and has since been sister and matron of the Boston Hospital, lady superintendent of the Taunton and Somerset Hospital, and matron of the General Hospital, Tunbridge Wells.

GRIMSBY AND DISTRICT HOSPITAL.—Miss Crichton has been appointed matron. She was trained at St. Bartholomew's Hospital, London, and was staff nurse for two years, doing temporary sister's duties. She has also been sister at Haslar, night superintendent and assistant matron at the General Hospital, Birmingham, and is now assistant matron at the City Hospital, Sheffield.

HOSPITAL SAMARITANO, SAO PAULO, BRAZIL.—Miss Amy L. Bridge has been appointed matron. She was trained at the Liverpool "Stanley Hospital," and has since been sister at the Grantham Hospital, sister of the male medical and surgical wards, Burton-on-Trent, night superintendent at the East Sussex Hospital, Hastings, and sister at the Women's Hospital, Soho Square, London.

NEWPORT AND MONMOUTHSHIRE HOSPITAL. — Miss Alice M. Jones has been appointed staff nurse. She was trained at Marylebone Infirmary, London, and has since been charge nurse at the Eastern Fever Hospital, and staff nurse at the Jessop Hospital, Sheffield.

RADCLIFFE, RAMSBOTTOM, WHITEFIELD AND BURY ISOLATION HOSPITAL.—Miss Florence D. Lewis has been appointed matron. She was trained at Stockport Union Infirmary, and held the post of staff nurse for 12 months. She has since done private nursing for $2\frac{1}{2}$ years at Manchester and Surbiton, and has been charge nurse on the hospital ships, Dartford, at the isolation camp for small-pox, Edmonton, N., charge nurse at the isolation hospital, Hertford, and superintendent nurse at Canterbury Workhouse Infirmary. She holds the L.O.S. certificate.

ROYAL INFIRMARY, LIVERPOOL.—Miss S. E. M. McNeil has been appointed assistant lady superintendent. She was trained at the Dumfries and Galloway Royal Infirmary and St. Bartholomew's Hospital, London, where she was afterwards temporary assistant to home sister. She has also been second assistant matron at the Southwark Infirmary, East Dulwich.

SOMERSET HOSPITAL, CAPETOWN, SOUTH AFRICA.—Miss A. Sutton has been appointed sister. She was trained at Guy's Hospital, London. She has been attached to the Army Nursing Service Reserve for two years, and has since been doing private nursing.

WOOLWICH INFIRMARY, PLUMSTEAD.—Miss Mary Farmer has been appointed ward sister. She has just completed her three years' training in that institution.

### Echoes from the Outside World.

### Movements of Royalty.

The King and Queen, with the Princess Victoria, are spending the Ascot week at Windsor Castle. On Monday their Majesties received in St. George's Hall some 200 delegates of the International Telegraph Conference, who were introduced by the Postmaster-General. The King conversed for some time with Mr. Austen Chamberlain, and the Queen graciously bowed to the delegates and the ladies who accompanied them as she passed in and out among them. It was intended to have a garden party in the Castle grounds, but in consequence of the very wet weather this arrangement was abandoned, and tea was served to the visitors in one of the galleries. The Royal Review which was to have been held at Aldershot on Monday has been postponed till a date in July yet to be fixed.

### Revolution in Servia.

The King and Queen of Servia were assassinated at Belgrade on Thursday of last week. A body of troops surrounded the Royal residence, overpowered the guard, and forced their way into the Konak, or Palace. Those who opposed them were shot, notably General Petrovics, aide-de-camp to the King, who tried in vain to stop the intruders. Suddenly the electric light was turned off, and the Palace plunged into total darkness. Feeling their way, the conspirators climbed the stairs, and found the candles lighted in the ante-room to the King's apartments, which were empty. Seizing the lights, the search for the unfortunate King and Queen began, and was unsuccessful till a manservant of Queen Draga's was discovered, made to reveal the hiding-place of his mistress, and promptly shot. Then the revolutionaries came upon the alcove which hid the door leading into the tiny chamber—a dressing-room—where the fugitives had concealed themselves. As the door was locked, it was smashed with an axe, and the insurgents rushed in. It is said that the older men only intended to make the King abdicate, with which request, bearing a paper to sign, they advanced towards him; but the younger men would not be restrained, opened fire at once, and both King and Queen fell riddled with bullets and slashed with sabres. The bodies were thrown out into the garden, where they lay for some time till they were wrapped in linen cloths, conveyed to the new Konak, and secretly buried on the following night. The graves are marked by two plain wooden crosses, one of which bears the word "Alexander," the other "Draga." The Royal pair were not the only victims. The houses of the Premier, the Minister of War, and other Ministers were surrounded, and they themselves slain, besides numerous soldiers. The same fate befell the Queen's brother, Nikodem Lunyivitza, whom the King is accused of trying to appoint as his successor. The revolution is stated to have been organised by the Army, who objected to this nomination, and also personally hated Queen Draga.

### The Destroyed Dynasty.

King Alexander was the last of the dynasty of the Obrenovitchs founded by Milosh, who caused Kara George, the "Black George," and founder of the Karageorgevitch family, to be assassinated because he was a rival in the people's favour. He was succeeded by his son Michael, who was assassinated in the royal deer park. His successor was Prince Milan, who gave Servia its first constitution. He was notorious for the bad life he led, and was divorced by his wife Natalie. In 1889 he was forced to abdicate, and his son Alexander—who has just met with so terrible a fate—was proclaimed king in his stead under a regency, at the age of 13. It was arranged that Alexander should attain his majority when he became 19, but when he was only 16 he took the law into his own hands, invited the regents to dinner one night, drew a revolver in the course of the meal, proclaimed himself of age, and imprisoned the regents for the night, next day taking a few Moderate Radicals for his advisers. In 1900 he announced his approaching marriage to Madame Draga, a widow twelve years his senior, who had acted as lady-in-waiting to his mother. There was much opposition to the match, but Alexander declared that a king no less than a peasant, should be allowed to wed as his heart dictated. The Servians were further incensed when six months later, after the King had announced that the birth of an heir to the throne was imminent, it was found that the Queen had misled him. From that time forth things went from bad to worse: the King came into direct conflict with his Cabinet, and the dislike of the Queen was openly shown; but such an awful termination was never anticipated in Europe, and has, of course, caused an immense sensation.

### The New King of Servia.

The day after the murders the conference of Senators and Deputies resolved that the Constitution of 1888 should be put in force, and that Prince Peter Karageorgevitch—a direct descendant of the "Black George" whom Milosh caused to be assassinated—should be unanimously elected King. The Prince was then in Geneva, where he received the news, accepted the crown, and stated that he would bear the name of Peter I. In his proclamation he thanked the Servian people, who had thus shown a desire to revert to the traditions of its ancestors, and gave his word that he would bury in oblivion the last 40 years, and entertain no ill feeling against those who opposed him. King Peter is 59 years of age and a widower with two sons. On Monday the Provisional Parliament assembled in the Konak, the President thanked the Army for "its heroic conduct," and then proceeded to confirm the election of the new King by personal voting.

### French Governesses' Home.

On Saturday afternoon, in pouring rain, Princess Henry of Battenberg opened the new home arranged and furnished in connection with the Association of French Governesses in England. It is situated at 18 Lancaster Gate, Hyde Park, and is under the direction of Madame Lauraint. At present there are 24 beds, some in single rooms, some in dormitories containing three or four beds; but, if needed, additional sleeping accommodation for six persons can be added. Governesses of all creeds are admitted—members seeking employment or who wish a rest for the holidays, as well as some who have daily engagements—the charges ranging from 18s. to £1 1s. a week. The reception-rooms are well furnished, and the electric light has been installed. Each applicant must have the necessary French diplomas, and furnish excellent references both with regard to herself and family. English governesses can be accommodated when the home is not full. The Princess was received by the French Ambassador, and he thanked her in the name of the French colony for the honour she had done them by her presence. The home was endowed, he stated, with the profits of the floral *fête* at the French Bazaar last year, when the Princess also favoured them with her presence.

### A Research Fellowship for a Lady.

It is announced that a Research Fellowship at Somerville College, Oxford, has been awarded to Miss Evelyn Johnson. Miss Johnson was educated at the Church of England High School, Graham Street, Eaton Square, London, and at Lady Margaret Hall, Oxford. She took a first class in the Honours School of Modern History at Oxford in 1901, and was the only woman placed in the First Class in that year. Since then she has held the post of secretary at the Graham Street High School.

### A Book and its Story.

NEW NOVEL BY A NEW AUTHOR.*

"How sour sweet music is
When time is broke and no proportion kept!
So is it with the music of men's lives."
*Richard II.*, Act v., Scene 5.

THE above lines give the source from which Mr. Carlyle has taken the title of his novel. It is not a particularly happy one, and those of us who select our books by their titles, may, perhaps, for this reason, pass over "Sour Music." In that case it will be a distinct loss, for the story is interesting and written with a force and directness that keeps the reader alert.

Mark Castlehaugh's character is a fine one. After long years of absence, "knocking about as a citizen of the world," he arrives in England from Egypt, and being a man without family ties, goes north to pay a long promised visit to an old friend, James Corday. A lonely, and self-centred man, Mark Castlehaugh found himself at thirty-five heart whole. The affection he had for his friend was one into which the memories of boyhood and youth were woven with an intensity that absence and a natural reserve had tended to deepen. Around "Corday's Sheiling" romantic memories hung, in which its owner, and his motherless little daughter, Marian, moved as dim but attractive figures. Marian was Mark's goddaughter; he had last seen her as a child of seven, and he was filled with excitement at the prospect of meeting her again. He had kept in touch with her through letters, and gifts that were chosen with much care.

Having arrived by an earlier train than the one by which he was expected, he made his way on foot to his destination. "Suddenly, as people do when they climb a hill, he came within sight of Corday's Sheiling. There it stood, its chimneys and gables outlined against the darkening sky, the yellow glow of fire-light and candle-light illumining some of the windows. It stood as the station-master had said, upon the summit of the fell. Turn which way the observer might, below him, on every side, he would see hills and valleys, the hills covered with trees, the valleys marked off by hedges into fields of arable and meadow land. Far to the north lay a silver streak of sea, this silver streak widening in the south to an open bay, this in daylight; at night, the electric lights of a town shone as starpricks. . . . But this vast stretch of country could not hold his attention, for was not this Corday's Sheiling at last! How often had he pictured his coming home to it!" When he arrived at the house no one greeted him. His friend had driven to the station for the later train and he waited in silent expectation for the first glimpse of the little goddaughter now grown to womanhood. The room into which he had been shown was wainscotted with dark wood, the floors were polished, an oval table was laid for three persons, lighted by candles with red shades. An oak press, a grandfather's clock, flowers in pots, red geraniums and white begonias in full bloom standing on the window-sill, china bowls filled with heather, pine branches and bracken, gave a sense of quaint homeliness and refinement. Before the girlish chatelaine makes her appearance he is first aroused by the tapping of high heels on the uncarpeted stairs preceded by something aimed at, but falling short of him, accompanied by light laughter. "It was a rose, and Mark Castlehaugh's head turned sharply at the sound of the clinking glasses among which it had fallen; he snatched it up, and, putting it to his lips, looked at the elfin eyes which now shone down at him over the balustrade."

Marian Corday was standing above him. "Why do you not come and shake hands with me?" she said.

The vision that greeted him was that of a girl slightly above the middle height, her dark brown hair coiled high on her head adding to her height. She had put on a fancy dress of her mother's, and appeared in stiffened bodice and farthingale, a cluster of roses pinned upon the bosom of her dress, the upper robe of sea-green flowered silk held aside with one hand from the white satin petticoat, beneath which one small red shoe appeared. "'You are not like the photograph you sent me,' he stammered, with a sense that he was babbling words which did not in the least express the things he desired to say. . . . 'You see you were a little girl when last I saw you ten years ago,' Mark Castlehaugh went on, still saying the things that he had no desire to say, while the things he wanted to say had fallen into hopeless confusion at the back of his mind. 'Yes, I was a little girl, only seven years old when you went out to Egypt.' Then the speaker added somewhat inconsequently, 'Because you were my godfather, I was so proud of you.'" Then follows a scene in which the game of cross purposes is played unconsciously. Mark Castlehaugh had come home with the intention of asking Marian to be his wife. Her father wished it. He had prepared the way by his gifts and letters, one of the more recent ones being a ring of precious stones whose initial letters formed the word "Dearest." "'See,' continued the girl, 'I am wearing the rings you sent me.' She stretched out her slender hands for inspection. 'That is my favourite.' She pointed to one. 'I remember it I sent it on your seventeenth birthday.'" Poor Mark blunders on through this first meeting, seeing the impossibility of conveying to Marian any sense of the love that has sprung to life at the sight of her; seeing, too, that she regards him as her very dear friend and godfather merely, so that he succeeds, in his embarrassment, in making her think that he is in love with another woman. Marian asks how long he will be at home. "'I am not going back to Egypt.' 'Not going back! That is glorious! After father there is no one in the world I care for but you.'" She clasped her hands, her face radiant, her voice excited. Mark continues. . . . "'I am to be in the London house. It is less salary, but I took it believing that I was going to be—settled, you understand.' . . . He paused—— 'I believed I was going to be married. . . . I believed, I thought'—stammered Mark Castlehaugh—'but my plans are changed since coming to England. I am not going to be married.' 'Why are you not going to be married?' came the youthful voice, sharply. 'Because—I made a mistake, that is all.' . . . 'Do you really care very much for this woman you were going to marry? Do you—well, do you love her?' There was a pause for fully a minute and then Mark Castlehaugh spoke. 'Yes, I love her,' he said, in a low voice. . . . 'Are you sorry that she does not love you?' 'Yes, I'm sorry.' 'Godfather?' 'Yes, Marian.' 'I am still your sweetheart godchild, and you still care for me—you still love me?' Perhaps a little unsteadily came the answer. 'Yes, you are still my sweetheart godchild—and—and I still love you.'" Then she directs him to go and meet her father. "'The carriage drive winds about until you come to the gate leading out upon the main road three quarters of a mile away. . . . Good-bye, good-bye, Godfather, till dinner-time. I am so glad you have come back.'" The working out of Mark Castlehaugh's fight with himself is particularly able. The story has real pathos, with an underlying element of tragedy. It is well worth reading.

* "Sour Music." By J. Newman Carlyle. 1 vol. Price 6s. (Publishers, A. and C. Black, London.)

### For Reading to the Sick.

"YET WILL I BE TO THEM A SANCTUARY."

THE summer sun was setting in the west,
And all the hills were radiant with the glow;
There passed before it, sinking to his rest,
A little bird, reluctantly and slow;
Long lingering in the light, he sought his nest
With measured flight, as being loath to go.

Pale and short-lived the winter sun declined,
'Mid watery clouds that gathered stormily;
When battling with his wings before the wind,
A weary songster sought his sheltering tree;
Swiftly he sped, as though in haste to find
The blessed haven where he fain would be.

Lord, so my heart, when earthly hopes are bright,
Waits lingeringly upon the homeward road;
But when all joy is hidden from my sight,
Save the sweet peace Thy mercy has bestowed,
I leave the world, and wing my eager flight
Swift as an arrow, to find rest in God.

—*Anon.*

---

"The temple of God is holy, which you are."

This is a truth of the spiritual life that speaks to us of the mercy and tenderness of our heavenly Father, "who remembereth our frame, and knoweth that we are but dust." It should fill every soul with joy and encouragement, for it speaks of the easy ways of Divine love, and tells us that the path to heaven is the path of little daily duties done perfectly, with the pure intention of giving glory to God. It applies to the sick as well as to those in health. It throws a light on their pains of mind and body and bids them take heart, as it shows that their lives may be one beautiful sacrifice, rich with offerings most precious in the sight of the Lord. It should be the care of every Christian, in the first moments of his day, to gather together and offer to God on high the prayers, work, and sufferings of the day for His greater glory. Let nothing be lost, for all is of value. Collect with loving care every fragment and offer it to God.—*Anon.*

---

We want to make our hearts a shrine wherein God may dwell abidingly. In society, in "the daily round, the common task," God must not be forgotten. Deep down in our hearts there should be always the thought of Him to calm, to check, to guide, to strengthen, to satisfy. If our minds are Christ-like, our deeds and words will be likewise. Surely the tongue would "refrain from evil," the lips "speak no guile," the life be pure and holy, were there the habitual recollectedness which comes of communion with Him. We can only win this grace by prayer and long practice.—*R. W. Randall.*

---

Be kind to our Darkness, O Fashioner, dwelling in Light,
And feeding the lamps of the sky;
Look down upon this one, and let it be sweet in Thy sight,
I pray Thee, to-night;
Oh watch whom Thou madest to dwell on its soil,
Thou Most High!

*J. Ingelow.*

### Notes and Queries.

FOR REGULATIONS SEE PAGE 136.

*Massage.*

(108) I have been practising as a masseur in New York and now wish to obtain work in London. I should be much obliged for any information which you could give me to attain this end. The names of any hospitals, sanatoria, or agencies where registers are kept would be useful.—*F. C.*

You might apply to the Manager, the Hamilton Association for Providing Trained Male Nurses, 75 Park Street, Grosvenor Square, W.

*Night Duty.*

(109) In the event of the night superintendent being absent, can the day superintendent nurse compel one of the day charge nurses to take night duty? What would be the penalty if she refused?—*Inquirer.*

Each hospital makes its own arrangements, and nurses who cannot obey orders should resign.

*Stewardess.*

(110) What are the duties of stewardess on board ship? Is a certain amount of hospital training essential? Are there any ships which employ trained nurses as stewardesses?—*F. E. B.*

The stewardess looks after the lady passengers, and, although a knowledge of sick nursing may be useful, it is not necessary. It is difficult to obtain a post as stewardess, though hospital training is a recommendation.

*Collecting Box.*

(111) Will you kindly tell me where I could purchase a collecting box to place on a dog's back to collect subscriptions for the Nursing Association of this place? I think it would help to bring the association before the notice of the factory hands.—*Queen's Nurse.*

Perhaps Messrs. Garrould, Edgware Road, W., could help you.

*Epileptics.*

(112) Will you kindly tell me the addresses of the homes or colonies for epileptics?—*District Nurse.*

The National Society for the Employment of Epileptics, the Secretary, 12 Buckingham Street, Strand, W.C.; the Home for Epileptics, Maghull, near Liverpool; the Hon. Secretary, 2 Exchange Street East, Liverpool; and the Meath Home of Comfort, Godalming.

*Hospital Training.*

(113) I am very anxious to become a nurse, but, as I am only 19, will you kindly tell me if there is any hospital where probationers are received at that age without a premium?—*E. W.*

In the "Nursing Profession: How and Where to Train" (Scientific Press) you will find the names of one or two children's hospitals where young probationers are received.

*Dental Surgery.*

(114) Will you kindly tell me if there is a dental school in London where ladies are trained as dentists, and if you have any idea what the fees would be?—*Nurse G. P.*

There is no dental school in London where ladies can be trained as dentists.

*Hospital.*

(115) Is the London Temperance Hospital a recognised training school for nurses?—*A. Z.*

Certainly it is.

*Vaccination.*

(116) Can you tell me if hospital nurses are allowed to vaccinate at any time, or under any circumstances?—*Nurse M.*

There is no law against it; but vaccination is a doctor's duty, and a nurse would be most ill-advised to usurp it.

*Home.*

(117) Can you kindly tell me of a home in the South of England where a nurse who has been ordered rest and change for some time could be received for a small sum of money?—*M. H.*

Write to the Hon. Secretary, Home of Rest for Nurses, Almondsbury Memorial Institute, Bristol.

---

**Standard Nursing Manuals.**

"The Nursing Profession: How and Where to Train." 2s. net; 2s. 4d. post free.

"Nursing: Its Theory and Practice." (Revised Edition). 3s. 6d. post free.

"Surgical Ward Work and Nursing." (Revised Edition). 3s. 6d. net.; 3s. 10d. post free.

"Practical Handbook of Midwifery." (New Edition). 6s. net; 6s. 3d. post free.

"Notes on Pharmacy and Dispensing for Nurses." 1s. post free.

"Fevers and Infectious Diseases." 1s. post free.

"The Art of Massage." (New Edition). 6s. post free.

